# Dataset of botulinum toxin A influence on interleukins under neuropathy

**DOI:** 10.1016/j.dib.2016.11.023

**Published:** 2016-11-15

**Authors:** Magdalena Zychowska, Ewelina Rojewska, Wioletta Makuch, Siro Luvisetto, Flaminia Pavone, Sara Marinelli, Barbara Przewlocka, Joanna Mika

**Affiliations:** a Institute of Pharmacology, Polish Academy of Sciences, Department of Pain Pharmacology, Krakow, Poland; b CNR, Institute of Cell Biology and Neurobiology, Rome, Italy; c IRCCS, Santa Lucia Foundation, Rome, Italy

**Keywords:** Neuropathic pain model, Botulinum neurotoxin A, Minocycline, Glia, Interleukins

## Abstract

Our data show that botulinum toxin A (BoNT/A) didn’t influence motor functions in naïve and CCI-exposed rats, but diminished the neuropathic pain-related behavior. The results indicate that BoNT/A administration diminished the spinal Iba-1 positive cells activation and, in parallel, downregulated IL-1beta. Moreover, we observed that in DRG the protein level of pronociceptive factors (IL-1beta and IL-18) decreased and antinociceptive (IL-10 and IL-1RA) factors increased. Additionally, our behavioral analysis shows that chronic minocycline treatment together with a single BoNT/A injection in CCI-exposed rats has beneficial analgesic effects (M. Zychowska, E. Rojewska, W. Makuch, S. Luvisetto, F. Pavone, S. Marinelli, B. Przewlocka, J. Mika, 2016) [1].

**Specifications Table**

**Table:** 

Subject area	Neuroscience
Specific subject area	Neuropathic pain
Data type	Figures, Table
Data acquisition	***1. BEHAVIORAL ANALYSIS***
	***1.1 Tactile stimulus***
	*1.1.1. Paw pressure test (Randall Selitto apparatus, Ugo Basile, Italy)*
	*1.1.2. Von Frey test (Dynamic Plantar Aesthesiometer, Ugo Basile, Italy)*
	***1.2. Thermal stimulus***
	1. *1.2.1 Paw withdrawal test (Hargreaves Analgesia Meter, Landing, NJ)*
	2. *1.2.2. Cold plate test (*Hot/Cold* Plate Analgesia Meter, Columbus Instruments, USA)*
	***1.3. Motor function and activity***
	*1.3.1. Motor function (Rota-rod apparatus, Ugo Basile, Italy)*
	*1.3.2. Exploratory activity test (Open field equipment)*
	***2. BIOCHEMICAL ANALYSIS***
	***2.1. Protein analysis***
	*2.1.1. Western blot technique*
Data format	One-way analysis of variance (ANOVA) and the inter-group differences were analyzed with Bonferroni׳s multiple comparison test and Student׳s t-test. GraphPad Prism software (ver. 7.0).
Experimental factors	The chemicals used were obtained from the following sources: • Minocycline hydrochloride (MC; 30 mg/kg, *i.p.*) from Sigma, USA • Botulinum neurotoxin serotype A (BoNT/A; 300 pg/paw, *i.pl*.), which was a kind gift from Prof. C. Montecucco and from Prof. O. Rossetto (University of Padova, Italy).
Experimental features	The experiments were carried out according to IASP rules. Neuropathic pain model - Chronic Constriction Injury (CCI model) of the sciatic nerve was performed according to Bennett and Xie (1988). Drugs intraperitoneal and intraplantar administration. The analgesic effect of minocycline and BoNT/A was measured in naïve an CCI-exposed rats. The biochemical study was performed to examine the drugs influence on CCI-induced changes on cytokines protein level in the DRG and spinal cord.
Data source location	Krakow, Poland
Data accessibility	Data within this article

**Data Value [1]**

• BoNT/A, in addition to altering neuronal function, can also influence non-neuronal cells, especially microglia.
• BoNT/A administration diminishes pro- and enhances anti-nociceptive interleukins in the spinal cord and DRG.
• The attenuation of non-neuronal cells activation may act as an additional factor in the long-lasting effects of BoNT/A on neuropathy.

## 1 Data [1]

**Scheme 1:**
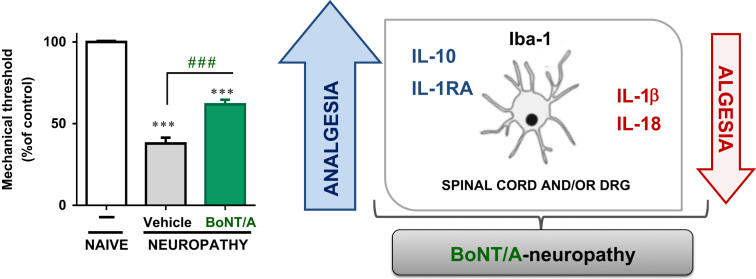
Graphical abstract showing the participation of pro- and anti-nociceptive interleukins in botulinum toxin A-induced (BoNT/A) analgesia in a rat model of neuropathic pain.

Single intraplantar BoNT/A injection significantly attenuated pain-related behavior. Moreover, minocycline enhanced its analgesic properties. The Western blot analysis suggests that CCI-induced the upregulation of IL-18, IL-6 and IL-1beta protein levels in the ipsilateral lumbar spinal cord and DRG, but no changes in IL-18BP, IL-1RA or IL-10 were observed. Interestingly, BoNT/A injection decreased spinal and/or DRG CCI-upregulated levels of IL-18 and IL-1beta and increased the levels of antinociceptive factors (IL-10 and IL-1RA) in the DRG **(**Scheme 1**).**

## 2 Experimental design, materials and methods

### 2.1 Animals

In our experiments we used male Wistar rats from Charles River (Sulzfeld, Germany).

### 2.2 Neuropathic pain model

Chronic Constriction Injury model [2], [3].

### 2.3 Drugs

• Minocycline hydrochloride (30 mg/kg intraperitoneal, Sigma, USA); scheme administration as described before in Rojewska et al. [4]
• Botulinum neurotoxin serotype A (300 pg of toxin in 0.9% NaCl per paw; a gift from Prof. C. Montecucco and from Prof. O. Rossetto, Padova, Italy); administration as described in Mika et al. [3]

### 2.4 Schemes for the study of repeated minocycline administration combined with a single BoNT/A injection in CCI-exposed rats

**Scheme 2:**
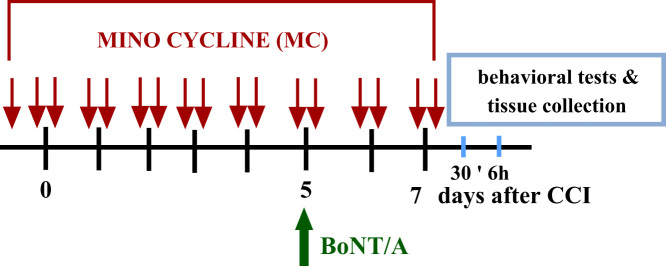
The way of drug administration (minocycline, MC; botulinum toxin A, BoNT/A) for behavioral and biochemical analysis after chronic constriction injury (CCI) to the sciatic nerve.

MC (30 mg/kg) was administered pre-emptively via *i.p.* injection 16 h and 1 h before CCI and then twice daily for seven days, referred to throughout the manuscript as repeated treatment. We had decided on this administration schedule, since microglial cell inhibitors reduce the activation of these cells more efficiently when the inhibitor is administered before an injury. On day 5 following CCI, the *i.pl.* injections of 20 μl of saline (0.9% NaCl) or BoNT/A (300 pg of toxin in 0.9% NaCl per paw) were performed. Next, on day 7 following CCI (two days after BoNT/A administration) and 30 min after the last injection of minocycline, behavioral tests (von Frey and cold plate tests) were performed. Tissue collection (spinal cord/DRG) was performed 6 h after the last administration of minocycline **(**Scheme 2**).**
