# Supplementary material for: Dataset of botulinum toxin A influence on interleukins under neuropathy
Source: Data Brief. 2016 Nov 15;9:1020–3. doi: 10.1016/j.dib.2016.11.023 (PMC5122696; doi:10.1016/j.dib.2016.11.023)
Supplement: Supplementary file 1 — Supplementary material [file mmc1.docx]

**
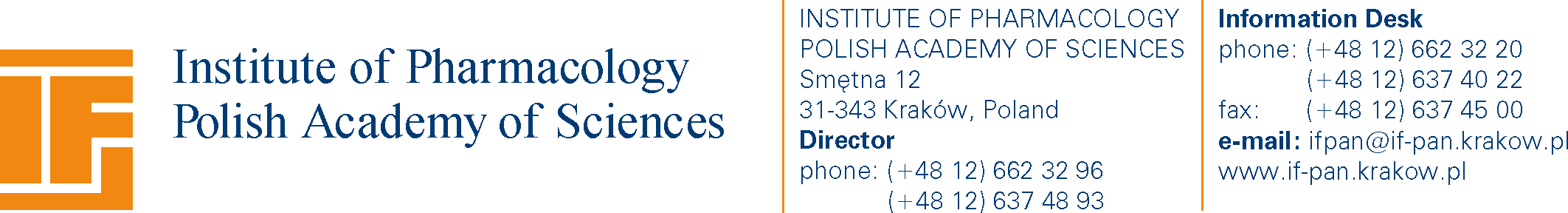
**

Dear Editors,

I am resubmitting a manuscript entitled: **“DATASET OF BOTULINUM TOXIN A INFLUENCE ON INTERLEUKINS UNDER NEUROPATHY”** by Magdalena Zychowska, Ewelina Rojewska, Wioletta Makuch, Siro Luvisetto, Flaminia Pavone, Sara Marinelli, Barbara Przewlocka and Joanna Mika through the journal’s online manuscript submission system.

**The present results is no conflict of interest for the co-authors of this paper.**

Sincerely yours,

Prof. Joanna Mika

Department of Pain Pharmacology

Institute of Pharmacology

Polish Academy of Sciences

12 Smetna Street, 31-343 Cracow, Poland

Phone: (48-12) 6623240; Fax (48-12) 6374500

e-mail: [joamika@if-pan.krakow.pl](mailto:joamika@if-pan.krakow.pl)
